# The neuropeptide neuromedin U promotes autoantibody-mediated arthritis

**DOI:** 10.1186/ar3732

**Published:** 2012-02-07

**Authors:** Sindhuja M Rao, Jennifer L Auger, Philippe Gaillard, Ralph Weissleder, Etsuko Wada, Richard Torres, Masayasu Kojima, Christophe Benoist, Diane Mathis, Bryce A Binstadt

**Affiliations:** 1 Center for Immunology and Department of Pediatrics, University of Minnesota, Medical Biosciences Building, 2101 6th St SE Minneapolis, MN, 55414, USA; 2 Department of Medicine, Division of Rheumatology, Washington University, 660 South Euclid Avenue, Campus Box 8118, St Louis, MO, 63110 USA; 3 Biostatistical Design and Analysis Center, Clinical and Translational Science Institute, University of Minnesota, 717 Delaware St. SE, Minneapolis, MN, 55414, USA; 4 Center for Systems Biology, Massachusetts General Hospital and Harvard Medical School, 185 Cambridge Street, Suite 5.210, Boston, MA, 02114 USA; 5 Department of Degenerative Neurological Diseases, National Institute of Neuroscience, NCNP, 4-1-1 Ogawa-Higashi, Kodaira, Tokyo 187, Japan; 6 Regeneron Pharmaceuticals, Inc, 777 Old Saw Mill River Road, Tarrytown, NY, 10591, USA; 7 Department of Molecular Genetics, Institute of Life Sciences, Kurume University, 1-1 Hyakunen-kohen, Kurume, Fukuoka 839-0842, Japan; 8 Department of Pathology, Harvard Medical School, 77 Avenue Louis Pasteur, Boston, MA, 02215, USA

## Abstract

**Introduction:**

Neuromedin U (NMU) is a neuropeptide with pro-inflammatory activity. The primary goal of this study was to determine if NMU promotes autoantibody-induced arthritis. Additional studies addressed the cellular source of NMU and sought to define the NMU receptor responsible for its pro-inflammatory effects.

**Methods:**

Serum containing arthritogenic autoantibodies from K/BxN mice was used to induce arthritis in mice genetically lacking NMU. Parallel experiments examined whether NMU deficiency impacted the early mast-cell-dependent vascular leak response induced by these autoantibodies. Bone-marrow chimeric mice were generated to determine whether pro-inflammatory NMU is derived from hematopoietic cells or stromal cells. Mice lacking the known NMU receptors singly and in combination were used to determine susceptibility to serum-transferred arthritis and *in vitro *cellular responses to NMU.

**Results:**

NMU-deficient mice developed less severe arthritis than control mice. Vascular leak was not affected by NMU deficiency. NMU expression by bone-marrow-derived cells mediated the pro-arthritogenic effect. Deficiency of all of the known NMU receptors, however, had no impact on arthritis severity and did not affect the ability of NMU to stimulate intracellular calcium flux.

**Conclusions:**

NMU-deficient mice are protected from developing autoantibody-induced inflammatory arthritis. NMU derived from hematopoietic cells, not neurons, promotes the development of autoantibody-induced inflammatory arthritis. This effect is mediated by a receptor other than the currently known NMU receptors.

## Introduction

Neuromedin U (NMU) is an evolutionarily conserved short neuropeptide with multiple physiologic effects. Named for its ability to induce uterine contraction, NMU has also been reported to play roles in metabolic and feeding regulation, pain perception, bone remodeling, blood pressure and contraction of smooth muscle in a variety of organs. NMU is widely expressed, with highest levels in the central nervous system and gastrointestinal tract [1]. NMU has not been detected in the circulation, suggesting that it acts primarily as a neurotransmitter and/or that it is short-lived [2].

Two NMU receptors have been identified, NMUR1 and NMUR2 [1,3]. Both of these are G-protein-coupled receptors with seven transmembrane domains. In most species studied, including the mouse, NMUR1 is widely expressed, predominantly in the gastrointestinal tract and also in immune cells, whereas the expression of NMUR2 is limited to the central nervous system. Binding of NMU to either receptor results in the elevation of intracellular calcium [4].

Several immunostimulatory activities have been attributed to NMU. In a mouse Th2 cell clone, stimulation with NMU led to intracellular calcium flux and the synthesis and release of IL-4, IL-5, IL-6, IL-10 and IL-13 [5]. More recently, attention has focused on the role of NMU on cells of the innate immune system. NMU induced calcium flux in, and degranulation of, mast cells and was required for mast-cell-mediated inflammation triggered by local injection of complete Freund's adjuvant [6]. In a mouse model of asthma, NMU activated eosinophils [7]. Furthermore, NMU could augment lipopolysaccharide-induced IL-6 production by macrophages [8]. These findings suggest that NMU might be an important driver of inflammatory diseases.

Arthritis can be induced by injecting serum from K/BxN T cell receptor (TCR) transgenic mice into normal mice, reflecting the high concentrations of arthritogenic autoantibodies recognizing glucose-6-phosphate isomerase (GPI) in the K/BxN arthritis model. The development of serum-transferred arthritis depends on innate immune cells, such as neutrophils and mast cells (although this cell type is under debate) as well as platelets, activating Fc receptors, the alternative pathway of the complement system, and cytokines [9-17]. Here, we utilized the K/BxN serum transfer model to test the hypothesis that NMU promotes inflammatory arthritis. We also investigated the cellular source of NMU during the development of arthritis and which of the NMU receptors mediate its pro-inflammatory effects.

## Materials and methods

### Mice

Mice with a targeted deletion of the gene encoding NMU (*Nmu^tm1Mko^*), NMUR1 (*Nmur1^tm1Rtor^*) and NMUR2 (*Nmur2^tm1Rtor^*) or NTSR1 (*Ntsr1^tm1Hmno^*) on the C57BL/6 (B6) background have been described [18-20]. The nomenclature for the targeted alleles is obtained from Mouse Genome Informatics [21]. Non-obese diabetic and B6 mice were obtained from Jackson Laboratory, Bar Harbor, ME, USA. KRN TCR transgenic mice were bred in house. Mice were maintained in specific-pathogen-free colonies at Harvard Medical School or the University of Minnesota, under protocols approved by the Harvard Medical Area Standing Committee on Animals or the University of Minnesota's Institutional Animal Care and Use Committee.

### Arthritis induction and related studies

K/BxN serum-transferred arthritis was induced and monitored as previously described [22]. Unless otherwise indicated, 150 μL of serum obtained from eight-week-old K/BxN mice was injected intraperitoneally into 6- to 8-week-old recipients on days 0 and 2. The arthritis scoring, measurement of ankle thickening and determination of anti-GPI immunoglobulin G (IgG) titers were performed as previously described [23]. For arthritis scoring, each paw was assigned a score of 0 (no arthritis) to 3 (maximum severity), resulting in a total range of 0 to 12 for an individual mouse. Mast cells were identified by toluidine blue staining, as previously described [11]. Vascular leak studies and generation of bone-marrow chimeric mice were performed as previously described [22]. Complete blood counts were performed using a Hemavet 950FS (Drew Scientific, Dallas, TX, USA).

### Calcium flux

Following lysis of red blood cells, splenocytes were resuspended in Roswell Park Memorial Institute medium plus 10% fetal bovine serum, and were loaded with Indo-1 AM (10 μg/mL; Invitrogen, Grand Island, NY, USA) for 30 minutes at 37°C. The cells were washed and resuspended at 5 × 10^6 ^cells/mL. Following the addition of indicated concentrations of rat NMU-23 (sequence YKVNEYQGPVAPSGGFFLFRPRN; GenScript, Piscataway, NJ, USA) or 1 μg/mL ionomycin (MP Biomedicals, LLC, Solon, OH, USA) in 12.5% dimethyl sulfoxide in water, the cells were analyzed on an LSRII flow cytometer (BD Biosciences, San Diego, CA, USA). Data were analyzed using FlowJo software (Treestar, Ashland, OR, USA).

### Statistical analysis

Statistical differences between the mean values for groups were calculated using an unpaired Student's two-tailed *t *test. Arthritis severity scores were compared with a repeated-measures analysis of variance. For experiments involving more than two groups, a post-hoc Tukey's multiple comparison test was used. *P *values < 0.05 were considered significant. Analyses were performed with SPSS 17.0.

## Results

**Figure 1:**
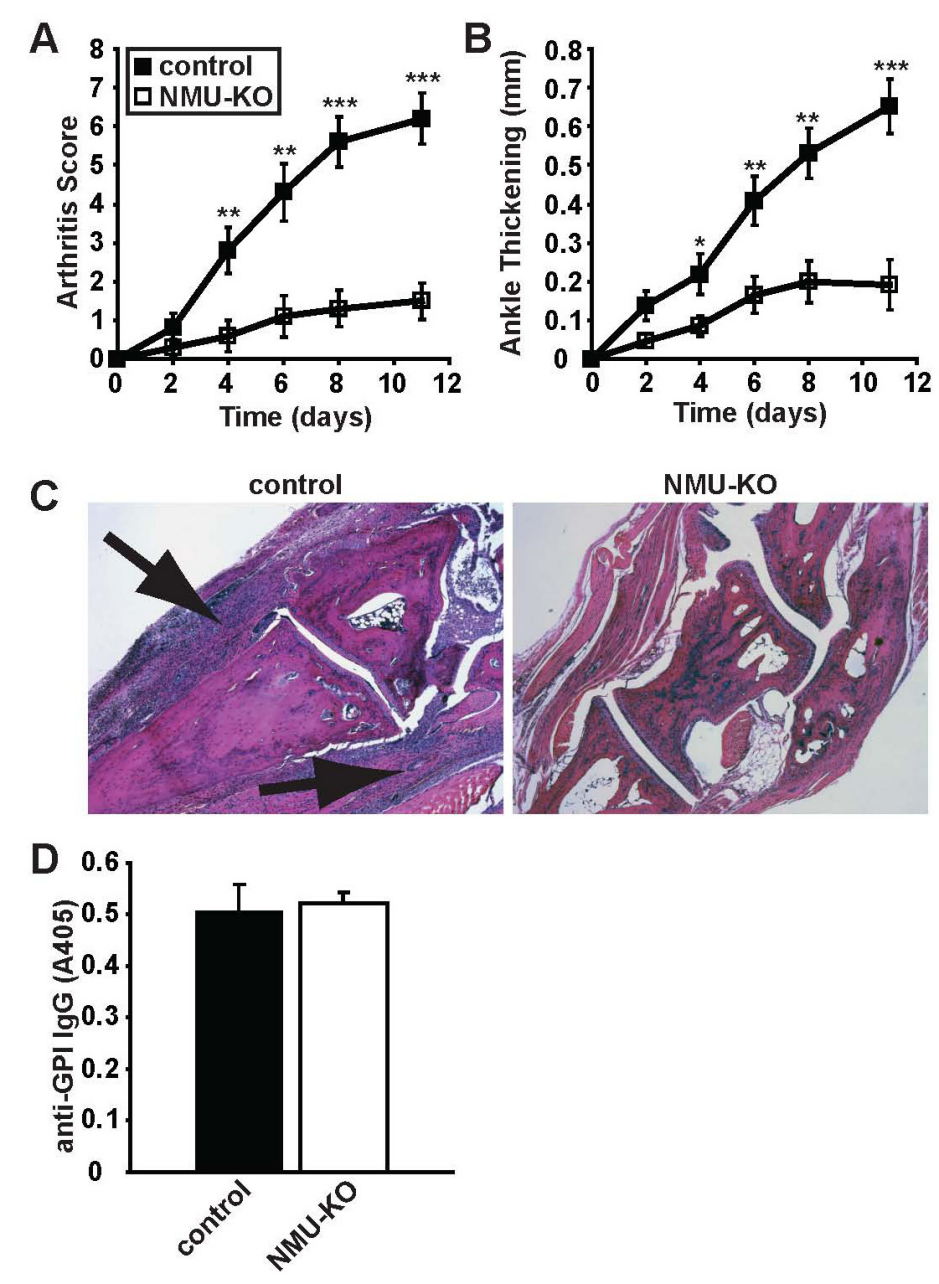
**NMU promotes autoantibody-mediated arthritis**. Serum from arthritic K/BxN mice was injected into NMU-KO mice or littermate control mice on days 0 and 2. The development of arthritis was assessed using **(A) **arthritis scores and **(B) **ankle thickening measurements. NMU-KO mice had significantly lower arthritis scores (*P *< 0.001, repeated-measures ANOVA) and ankle thickening (*P *= 0.001, repeated measures ANOVA), leading to the conclusion that arthritis severity increases more quickly in wildtype mice relative to the NMU-KO mice. Data plotted are means ± SEM from 10 mice per group compiled from three independent experiments. *P*-values for individual timepoints calculated using two-tailed Student's T-test are also depicted: *P < 0.05; **P < 0.01; ***P < 0.001. **(C) **Representative ankle sections from the indicated mice at the peak of arthritis severity were stained with H&E. Arrows indicate inflammatory infiltrates. Original magnification ×5. **(D) **Serum was collected from the mice at the end of the experiment, diluted 1:900, and assayed by ELISA for the presence of anti-GPI IgG. Data plotted are arbitrary ELISA absorbance values (means ± SEM) from seven mice per group compiled from two independent experiments; *P *= 0.78. ANOVA: analysis of variance; ELISA: enzyme-linked immunosorbent assay; H&E: hematoxylin and eosin stain; GPI: glucose-6-phosphate isomerase; IgG, immunoglobulin G; KO: knockout; NMU: neuromedin U; SEM: standard error of the mean.

We utilized *Nmu *gene knockout (NMU-KO) mice to investigate a potential role for NMU in the pathogenesis of autoantibody-mediated arthritis. After injection of arthritogenic serum from K/BxN TCR transgenic mice, NMU-KO mice developed less severe arthritis than littermate controls (Figure 1A, B). Histological examination of the ankles was consistent with the clinical scoring, with less inflammation present in the NMU-deficient mice (Figure 1C). We confirmed that NMU-KO and control mice maintained equivalent titers of the pathogenic anti-GPI IgG (Figure 1D). These results indicate that NMU promotes the development of autoantibody-induced arthritis.

**Figure 2:**
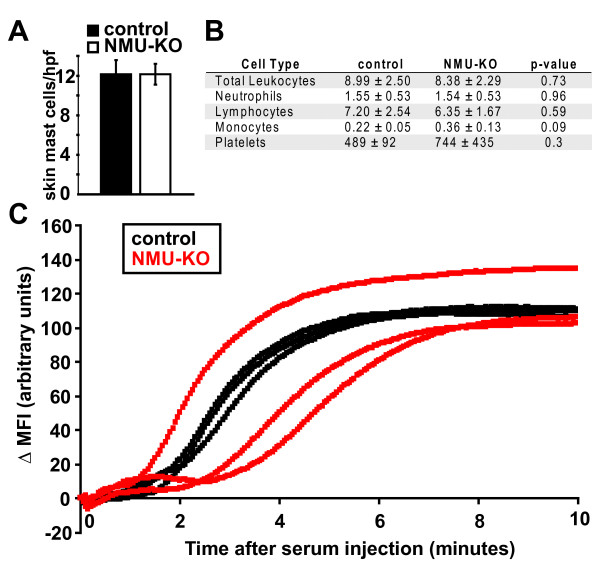
**NMU-deficient mice have mast cells, leukocytes, platelets and an intact early vascular response to arthritogenic antibodies**. **(A) **Mast cells were enumerated in sections of skin obtained from control B6 (*n *= 3) and NMU-KO (*n *= 4) mice and stained with toluidine blue. Cells were counted in five ×40 HPF per mouse and averaged. Plotted values represent the number of mast cells per HPF in the indicated groups (mean ± SEM). **(B) **Complete blood counts from control B6 and NMU-KO mice were performed. Numbers shown are × 10^9^/L ± SD, with four mice per group. **(C) **Serum from K/BxN mice was injected intravenously into NMU-KO mice (red) or littermate control mice (black). Vascular leak in the hindpaw was monitored by intravital confocal microscopic evaluation of extravasation of the intravascular tracer, Angiosense 680. Each line represents one mouse. B6: C57BL/6; HPF: high power field; KO: knockout; MFI; mean fluorescence intensity; NMU: neuromedin U; SD: standard deviation; SEM: standard error of the mean.

NMU has been shown to directly activate mast cells to produce vasodilation and plasma extravasation in response to complete Freund's adjuvant [6]. Similarly, we and others have shown that systemic injection of serum from K/BxN mice results in rapid induction of mast-cell- and neutrophil-dependent vascular permeability in the paws of mice [22,24,25]. Although some mast-cell-deficient mouse strains (W/W^v^) are protected from developing K/BxN serum-transferred arthritis [11,16] whereas others (W^sh^) are not [26,27], both the W/W^v ^and W^sh ^strains lack the early vascular response induced by administration of serum from K/BxN mice, verifying the mast-cell-dependence of this response [22] (BAB, RW, CB and DM, unpublished work). We therefore sought to determine whether NMU deficiency impacted these earliest events induced by K/BxN serum. We first verified that deficiency of NMU did not interfere with mast cell differentiation; mast cells were easily identified and present in equivalent numbers in the skin of B6 and NMU-KO mice (Figure 2A). In addition, the number and frequency of other leukocyte subpopulations and platelets were unaltered in the NMU-KO mice relative to controls (Figure 2B). Prior studies have shown that macrophages derived from NMU-KO mice produced normal levels of IL-1β and TNF, critical arthritogenic cytokines in K/BxN serum-transferred arthritis and also IL-12p40 following lipopolysaccharide stimulation. IL-6 production by NMU-KO macrophages was decreased relative to control macrophages, but IL-6 is dispensable for serum-transferred arthritis [8,10]. Similarly, mast cells from NMU-KO mice degranulated normally *in vivo *after the injection of NMU or substance P [6]. Consistent with these observations, we found that injection of K/BxN serum resulted in a similar vascular leak response in NMU-KO mice and wild-type littermates (Figure 2C). These findings indicate that the reduction in arthritis severity in NMU-KO mice was due neither to impaired differentiation nor a decreased inherent functional capacity of the main innate immune cell types that contribute to the multistep pathogenesis of arthritis in this model. In addition, the arthritis-promoting effect of NMU likely occurred at a timepoint after the initial vascular leak response.

**Figure 3:**
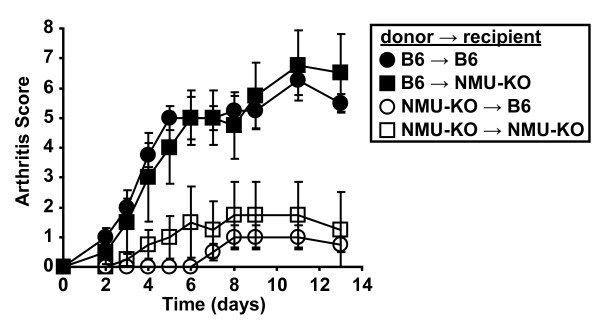
**NMU production by bone marrow-derived cells promotes autoantibody-mediated arthritis**. Bone marrow chimeric mice were generated in the four indicated combinations. Following bone marrow engraftment, serum from K/BxN mice was injected on days 0 and 2 and the development of arthritis was assessed at the indicated timepoints. Data plotted are means ± SEM and represent one of two independent experiments with a total of eight mice per genotype. B6→B6 versus NMU-KO→B6, *P *= 0.006; B6→B6 versus NMU-KO→NMU-KO, *P *= 0.022; B6→NMU-KO versus NMU-KO→B6, *P *= 0.007; B6→NMU-KO versus NMU-KO→NMU-KO, *P *= 0.027. There was no statistical difference between the two groups in which B6 mice were donors (*P *= 0.999) or between the two groups in which NMU-KO mice were donors (*P *= 0.880) (repeated-measures analysis of variance followed by post-hoc Tukey's multiple comparison test). B6: C57BL/6; NMU: neuromedin U; SEM: standard error of the mean.

NMU is best known as a neurotransmitter. However, other cell types, including hematopoietic cells, express NMU. Prior studies have not established whether NMU production by hematopoietic cells or stromal cells (for example, neurons) is responsible for its pro-inflammatory effects [6-8]. To address this question, we generated bone-marrow chimeric mice using wildtype B6 mice and NMU-KO mice in each possible donor:recipient combination. Following bone marrow engraftment, we induced arthritis with serum. Mice that had received wildtype bone marrow developed more severe arthritis than those that received NMU-KO bone marrow, irrespective of the NMU status of the recipient mouse (Figure 3). These findings establish that NMU expression by bone-marrow-derived cells, not neurons or other stromal cells, is responsible for the pro-arthritogenic activity of NMU.

**Figure 4:**
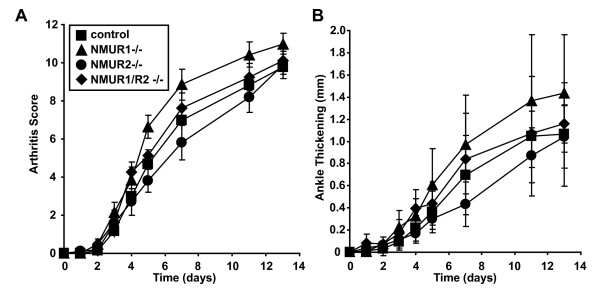
**NMUR1 and NMUR2 are dispensable for the induction of autoantibody-mediated arthritis**. Serum from K/BxN mice was injected into mice lacking NMUR1, NMUR2, both NMUR1 and NMUR2, or littermate controls animals on days 0 and 2. The development of arthritis was assessed by determination of **(A) **arthritis score and **(B) **ankle thickening. Data plotted are means ± SEM and are compiled from four experiments with eight to fourteen animals per genotype. For arthritis score (A), there were no statistical differences between the four groups. For ankle thickening (B), ankle thickening scores increased slightly more rapidly in the NMUR1-/- mice relative to the control mice (*P *= 0.042) and relative to the NMUR2-/- mice (*P *= 0.015). There were no statistically significant differences between the other groups of mice (repeated-measures analysis of variance followed by post-hoc Tukey's multiple comparison test). SEM: standard error of the mean.

Two main receptors for NMU have been identified, NMUR1 and NMUR2. The former is widely expressed, whereas expression of the latter is restricted essentially to the central nervous system. We utilized gene-knockout mice to determine which receptor mediates the pro-inflammatory activity of NMU. Mice lacking NMUR1, NMUR2 or both receptors developed arthritis in an essentially indistinguishable way from control mice (Figure 4). This observation suggests that another NMU receptor may be involved.

**Figure 5:**
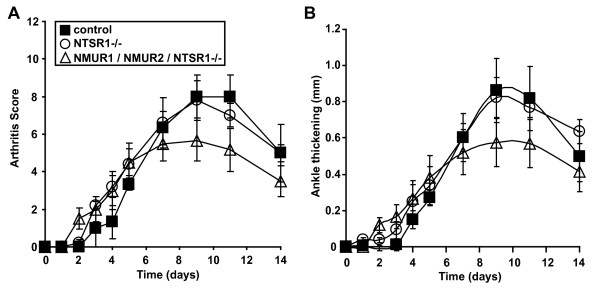
**Mice lacking all known NMU receptors remain susceptible to autoantibody-mediated arthritis**. Serum from K/BxN mice was injected into mice lacking NTSR1 or mice lacking NMUR1, NMUR2, and NTSR1 on days 0 and 2 (100 μL/injection). The development of arthritis was assessed by determination of **(A) **arthritis score and **(B) **ankle thickening. Data plotted are means ± SEM and represent one of three independent experiments with a total of eight to fifteen mice per group. There were no statistically significant differences between the groups (repeated-measures analysis of variance followed by post-hoc Tukey's multiple comparison test). NMU: neuromedin U; SEM: standard error of the mean.

The only other NMU receptor reported in the literature is a heterodimer of the neurotensin receptor 1 (NTSR1) and the growth hormone secretagogue receptor 1 b, identified in human lung cancer cells [28]. Therefore, we investigated the possibility that this receptor was responsible for the arthritis-promoting effect of NMU. *Ntsr1*-deficient mice developed serum-transferred arthritis equivalent to that of controls (Figure 5). We therefore bred mice lacking *Nmur1*, *Nmur2 *and *Ntsr1*. These mice, lacking all known NMU receptors, also remained susceptible to serum-transferred arthritis, even when given doses of serum lower than usual in an effort to accentuate any potential differences between the groups (Figure 5). This result was unexpected, and prompted us to perform additional *in vitro *studies to determine if cellular responses to NMU were intact in these triple-NMU-receptor knockout mice.

**Figure 6:**
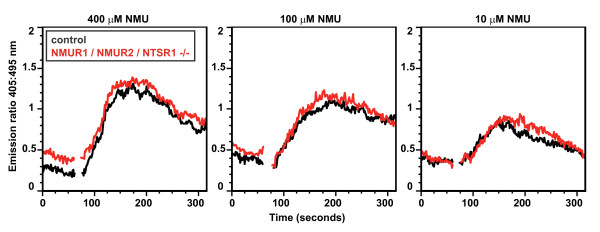
**NMU triggered calcium flux remains intact in splenocytes lacking all known NMU receptors**. Splenocytes from C57BL/6 mice (black) or mice lacking NMUR1, NMUR2 and NTSR1 (red) were loaded *in vitro *with the calcium sensitive dye Indo-1 and then stimulated with NMU at the 60 second timepoint at the indicated concentrations. Intracellular calcium flux was measured with flow cytometry and is plotted as the ratio of fluorescence emission at 405 nm (bound Indo-1) to fluorescence emission at 495 nm (free Indo-1) over time. Data are representative of three independent experiments. NMU: neuromedin U.

NMU stimulation of cells causes intracellular calcium flux. Stimulation of splenocytes from *Nmur1*/*Nmur2*/*Ntsr1 *triple-knockout mice with a range of concentrations of NMU provoked intracellular calcium flux indistinguishable from that induced in splenocytes from control mice, demonstrating that the splenocytes from these triple-knockout mice must utilize another mechanism to detect NMU (Figure 6). Considered together, our findings indicate that NMU can promote the development of autoantibody-mediated arthritis through a receptor distinct from the currently known NMU receptors.

## Discussion

Neuropeptides are increasingly recognized as potential contributors to inflammatory diseases. For instance, the neuropeptide substance P is involved in a variety of inflammatory conditions, including being upregulated in the inflamed synovium of patients with rheumatoid arthritis [29]. In addition, catecholamine-producing cells are present in the synovial tissue of mice and human patients with inflammatory arthritis [30]. Understanding how neuropeptides promote inflammatory diseases is expected to lead to new therapeutic approaches.

Based on a series of reports describing the pro-inflammatory properties of NMU [6-8], we tested the hypothesis that NMU can promote the development of autoantibody-mediated arthritis. Indeed, our results demonstrated that mice lacking NMU were protected from K/BxN serum-transferred arthritis. This protection was not due to impaired differentiation of the key innate immune cell types (that is, neutrophils, platelets and mast cells) implicated in this disease model. Nor did NMU interfere with the earliest vascular events provoked by injection of autoantibodies, but more likely at a later time. NMU can directly activate effector cells, such as mast cells, eosinophils and macrophages, to elicit a variety of cellular responses including degranulation, adhesion, chemotaxis and production of the pro-inflammatory cytokine IL-6 [6-8]. Therefore, it is likely that NMU acts on multiple cell types to elicit a variety of pro-inflammatory activities that drive the inflammatory arthritis in this model.

Although it is known that NMU can be produced by hematopoietic cells [8,31], most prior studies have focused on neurons as being the most likely source of pro-inflammatory NMU [6-8]. Our reciprocal bone-marrow transplantation experiments showed clearly that NMU derived from hematopoietic cells was critical for its pro-arthritogenic activity. Our findings are consistent with the finding that NMU is not a marker of peripheral sympathetic or sensory nerve fibers [3]. Similarly, our results support previous findings that substance P and neuropeptide Y are neuropeptides produced not only by neurons but also by immune cells [29,32], and that inflammatory arthritis is accompanied by a loss of sympathetic nerve fibers which appear to be replaced by synovial cells that synthesize catecholamines [30]. Thus, although neuropeptides are typically initially characterized based on their expression in neurons, their pro-inflammatory properties might be attributed instead to their expression by cells of the immune system, as we have shown. Therefore, the involvement of a neuropeptide in an inflammatory disease does not necessarily imply neuro-immune connections or neuro-immune cross-talk other than expression of similar neuropeptides by these cell types.

Additionally, although it is theoretically possible that NMU-deficient mice were protected from autoantibody-induced arthritis due to developmental or metabolic abnormalities in the mice, our finding that the protective effect of NMU deficiency was transferrable with bone marrow-derived cells suggests that such a scenario is unlikely.

Defining the receptors that a pro-inflammatory substance engages to elicit inflammation is important for the development of potential therapeutic agents. NMUR1 is widely expressed, and *Nmur1*-deficient mice have impaired contraction of certain smooth muscle tissues in the gastrointestinal tract [33]. NMUR2 is expressed primarily in the central nervous system, and *Nmur2*-deficient mice have been shown to have impaired responses to pain in some assays; in contrast, these pain responses were normal in *Nmur1*-deficient mice [20]. With respect to inflammatory responses, inflammation induced by the injection of complete Freund's adjuvant was normal in mice lacking both receptors [20]. Similarly, we have shown here that autoantibody-induced arthritis was unimpaired in these same *Nmur1/Nmur2*-deficient mice. We further have shown that the only other reported functional NMU receptor, a heterodimer of NTSR1 and the growth hormone secretagogue receptor 1 b, was also dispensable for the development of serum-transferred arthritis. Recent evidence, however, suggests that NMU-induced suppression of food intake and weight gain remained intact in mice lacking NTSR1, suggesting that NTSR1 may not serve as an NMU receptor *in vivo *[34]; this is consistent with our *in vitro *findings. It is theoretically possible that the related NTSR2 could serve as an NMU receptor, though this has not been demonstrated experimentally.

Our results suggest that an as-yet-unidentified NMU receptor is responsible for the pro-inflammatory effects of NMU. It is likely, based on the structure of the known NMU receptors, that this receptor will belong to a large family of seven transmembrane spanning G-protein-coupled receptors. It is of course possible that the expression of this putative additional NMU receptor is upregulated in the mice we studied here, as a physiologic compensatory mechanism for the absence of the known NMU receptors, and that its contribution to the pro-inflammatory activities of NMU is therefore apparent only in the absence of the known NMU receptors. It has been shown, for instance, that the expression of NMUR1 is increased in macrophages derived from NMU-KO mice, supporting the notion that such compensatory mechanisms might exist [8]. Further investigations such as the use of NMUR1- and NMUR2-specific short-acting antagonists and/or identification of this putative additional NMU receptor should help to clarify this point.

In summary, we have shown that mice genetically deficient in NMU expression were protected from developing autoantibody-induced inflammatory arthritis. In this model, bone-marrow-derived cells rather than neurons served as the critical source of NMU. The arthritogenic activity of NMU may be mediated via a receptor other than the currently defined NMU receptors. Our results indicate that targeting NMU or its receptors could be an attractive approach to therapeutic intervention in inflammatory arthritis, but also suggest that further delineation and definition of the key NMU receptors will first be required. More broadly, our findings demonstrate that neuropeptides expressed by immune cells can be important contributors to the pathogenesis of inflammatory arthritis.

## Conclusions

NMU produced by bone-marrow-derived cells promoted autoantibody-mediated arthritis in mice. The arthritogenic activity of NMU was mediated by a receptor other than the currently known NMU receptors. This study points to NMU as a new potential therapeutic target in inflammatory arthritis.

## Abbreviations

B6: C57BL/6; GPI: glucose-6-phosphate isomerase; IgG: Immunoglobulin G; IL: interleukin; KO: knockout; NMU: neuromedin U; TCR: T cell receptor; TNF: tumor necrosis factor.

## Competing interests

RT is an employee of Regeneron Pharmaceuticals. CB, DM and BAB have filed a United States patent application describing NMU as a therapeutic target in rheumatoid arthritis.

## Authors' contributions

SMR and JLA designed and performed the experiments and prepared and edited the manuscript. PG performed the statistical analysis. RW oversaw imaging experiments and edited the manuscript. EW provided *Ntsr1 *knockout mice and edited the manuscript. RT provided *Nmur1 *and *Nmur2 *knockout mice and edited the manuscript. MK provided NMU knockout mice and edited the manuscript. CB and DM designed and oversaw the experiments and edited the manuscript. BAB designed, performed and oversaw experiments, performed statistical analysis and wrote and edited the manuscript. All authors read and approved the final manuscript.
